# Intensive language training enhances brain plasticity in chronic aphasia

**DOI:** 10.1186/1741-7007-2-20

**Published:** 2004-08-25

**Authors:** Marcus Meinzer, Thomas Elbert, Christian Wienbruch, Daniela Djundja, Gabriela Barthel, Brigitte Rockstroh

**Affiliations:** 1 Department of Psychology, University of Konstanz, Universitätsstrasse 10, 78464 Konstanz, Germany; 2 Lurija Institute for Rehabilitation Research, Kliniken Schmieder, 78476 Allensbach, Germany

## Abstract

**Background:**

Focal clusters of slow wave activity in the delta frequency range (1–4 Hz), as measured by magnetencephalography (MEG), are usually located in the vicinity of structural damage in the brain. Such oscillations are usually considered pathological and indicative of areas incapable of normal functioning owing to deafferentation from relevant input sources. In the present study we investigated the change in Delta Dipole Density in 28 patients with chronic aphasia (>12 months post onset) following cerebrovascular stroke of the left hemisphere before and after intensive speech and language therapy (3 hours/day over 2 weeks).

**Results:**

Neuropsychologically assessed language functions improved significantly after training. Perilesional delta activity decreased after therapy in 16 of the 28 patients, while an increase was evident in 12 patients. The magnitude of change of delta activity in these areas correlated with the amount of change in language functions as measured by standardized language tests.

**Conclusions:**

These results emphasize the significance of perilesional areas in the rehabilitation of aphasia even years after the stroke, and might reflect reorganisation of the language network that provides the basis for improved language functions after intensive training.

## Background

Cerebrovascular stroke is a highly prevalent condition and the major cause of language impairment in adults. Immediately following a stroke about 38% of the affected population experience aphasia [1]. Spontaneous recovery is reported within the first six months after the event, while only minimal spontaneous improvements of language functions are expected after more than one year post-stroke [2]. Additional rehabilitation efforts have produced beneficial effects, as reported for speech and language therapy on the basis of different performance indices [3,4]. In accordance with recent progress in neurorehabilitation, which takes into account evidence of the brain's capacity for reorganization [5-7], intensive language training (several hours per week) seems to be the premise for substantial improvement of language functions in the chronic stage [8].

To date, the evaluation of impairment and recovery of function, including training-induced improvement in aphasia, has been based mainly on performance in neuropsychological tests. This is now being increasingly complemented by measures of brain function. Different mechanisms and time courses of recovery of language function after brain damage have been discussed. Hemodynamic imaging suggests the involvement of two mechanisms: (1) regression of diaschisis (reduced metabolism and function in areas connected with the damaged brain tissue, which have been cut off from essential input) and (2) functional reorganization of the neuronal networks involved in language processing. Regression of diachisis in perilesional and more distant regions have been shown to contribute to recovery of function particularly in early phases of the recovery process [9]. In contrast, "re"-recruitment (that is, reorganization) of perilesional areas of the left hemisphere [10] or reactiviation of left hemisphere network components [11,12] predict long-term recovery of language function. Moreover, recruitment of homotopic right-hemispheric areas may contribute to language recovery when the left-hemisphere language network components are permanently impaired [13]. However, it has been debated whether the recruitment of right hemispheric networks constitutes an additional potential for language processing or whether it is just a by-product of increased general activation. Others suggest this recruitment may even impair the recovery of left hemispheric areas, leading to a persistence of deficits [14].

Brain structures in the vicinity of structural lesions produce a larger amount of slow wave activity. This might be due to a loss of afferent input (e.g. from the lesion) or to a primary metabolic change within these perilesional areas [15].

These abnormal slow waves can be detected in the electroencephalogram (EEG) and, due to their focal generators, they can be localized using magnetic source imaging, a magnetencephalogram (MEG) based technique. In Abnormal Slow Wave Activity Mapping (ASWAM) [16], generators of abnormal slow waves are localized and mapped on to brain structures in order to identify areas that are active but incapable of normal function. A number of studies have demonstrated that focal slow waves indicate abnormality resulting from neurological damage such as contusions, tumors, or cerebrovascular stroke. In particular, abnormal slow wave activity in the delta-frequency range (1–4 Hz) has been found in areas adjacent to the structural lesion [17-19]. Since focal slow wave activity varies with changes in metabolism and blood flow due to the insult [19,20], it has been described as characteristic of a 'dysfunctional state' [21] of the neuronal tissue or a dysfunctional border zone with little ongoing information processing. In patients with brain tumors, this relationship between slow wave activity and metabolic changes was further elucidated by combining MEG and proton MR spectroscopic imaging [22]. A mild reduction of N-acetyl aspartate (NAA) and slight accumulation of lactate (Lac) was found in association with sources of focal slow wave activity in the border zones of the tumors, suggesting a border zone between seriously damaged and normal tissue with potential for re-recruitment in the course of the disease.

The mapping of abnormal slow wave activity can be used not only to identify dysfunctional neuronal networks, but also to track changes in the course of recovery or treatment. For instance, de Jongh et al. [17] reported increased focal delta activity in the MEG before and a reduction after resection of brain tumors. The utility of 'abnormal slow wave mapping' (ASWAM) in diagnostics, recovery, or treatment evaluation may be validated by covariation with neuropsychological measures. Lewine et al. [23] found a correlation between symptom resolution and MEG-slow wave reduction in patients with minor traumatic brain injury (TBI) and Hensel et al. [24] reported a decrease of EEG-delta amplitude and dipole strength parallel to spontaneous recovery of language functions across the first year post stroke in aphasia patients.

The present study employed ASWAM before and after intensive language training in aphasic patients. If ASWAM qualifies for the evaluation of treatment or training-supported rehabilitation in chronic aphasics, changes in the intensity and distribution of focally generated abnormal slow wave activity should vary with improvement of language function after a specific intervention. Aphasics were recruited from an ongoing project evaluating the effectiveness of an intensive language training program. This program combines the learning principles of shaping and the efficacy of concentrated training [25,6] while considering the principles of cortical reorganization [5]. In order to minimize any influence of spontaneous recovery on changes in the brain-function measure, only chronic aphasics were selected to participate either in 30 hours of Constrained-Induced Aphasia Therapy (CIAT) [25] or in 30 hours of massed model-based (MB) aphasia therapy [26]. All training sessions were scheduled within a two-week period.

It was hypothesized that (a) aphasics would display an increased density of slow wave generators in the damaged (left) hemisphere before training, (b) this density would be reduced in the perilesional zone following language training and (c) there would be an improvement of language functions as evaluated by a standardized language test (Aachen Aphasia Test Battery, AAT) [27].

## Results

### Language functions

The average test performance of the entire patient group increased after language training, as indicated by the AAT profile (t(27) = 9.85, p < 0.0001, paired t-test, two tailed). Similar improvements were found for the Token Test (t(27) = 6.10, p < 0.0001). The average improvement of the profile score was 2.9 ± 1.3 points and 6.1 ± 5.3 points on the Token Test (T-scores). Twenty-five of the 28 patients improved on at least one subtest (N = 19) or subscale (N = 6) of the AAT.

### Maximum delta activity

**Figure 1:**
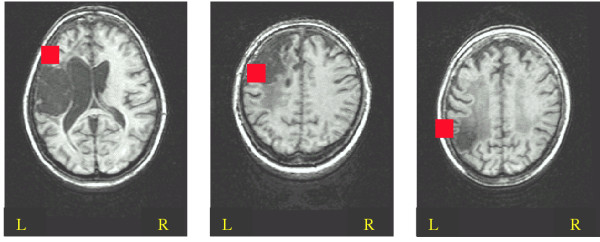
**Maximum delta activity in three representative subjects. **MEG superimposed on individual structural MRI of each patient: Localization of the voxel with maximum delta dipole density (red square) in three representative subjects (no.15, 25 & 28) located at the border zone of the structural lesion (l = left; r = right).

In 26 subjects the maximum activity of delta dipoles was found in the left hemisphere and in the vicinity of the structurally obvious lesion (as verified by structural MRT; see Figure 1 for three representative subjects). In one patient, the maximum delta activity was located in the right hemisphere anterior to the homologue of the lesion, a finding consistent across measurements. (The patient had a very mild amnesic aphasia, displayed the highest AAT profile score of the entire group [63.15] and showed the least amount of delta activity.) In another subject, the maximum delta activity was located at the posterior border of a large left fronto-temporal lesion due to an ischemic infarct of the middle cerebral artery in the first measurement. After training, the maximum delta activity was found in the right-hemispheric area anterior to the homologue of the lesion. Notably, both measurements showed that this patient had clusters of delta activity next to the lesion and its right hemispheric homologue. Left hemispheric Delta Dipole Density (DDD) decreased after training and increased in the right hemisphere, which might explain the shift of peak activity to the right.

The location of this delta focus remained stable across the two measurements (Rho: x-axis: .69, p < 0.0001, y-axis: .85, p < 0.0001, z-axis: .73, p < 0.0001). The coordinates of maximum delta dipole density were exactly the same in eleven patients, while maximum delta activity shifted by one voxel in one of the three cardinal planes in eight patients, and by more than one voxel in nine patients. (As emphasized above, one patient displayed a reversal in hemispheric lateralization after training).

### Hemisphere-specific average delta activity

**Figure 2:**
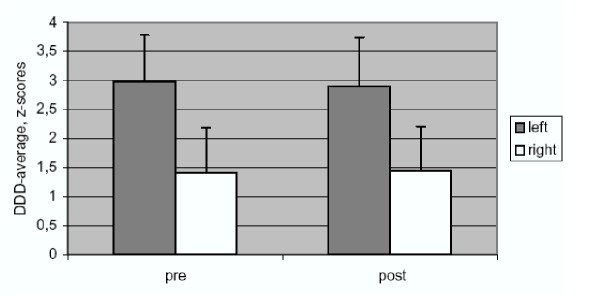
**Hemisphere averages before (pre) and after (post) intensive language training. **Average of voxels with high delta dipole density (DDD) in each hemisphere before and after therapy (all patients). It is notable that, at both measurements, clusters of high DDD are predominantly located in the left hemisphere. ANOVA revealed significantly higher DDD averages in the left hemisphere for both measurements.

Thresholds were significantly higher in the left hemisphere (F(1,54) = 49.03, p < 0.0001). Clusters of voxels with delta activity > 2 SD above the average DDD in a group of 25 healthy controls were found in 26 of the 28 patients in the left hemisphere before training. Such clusters were found in the right hemisphere in only 7 patients. In two patients only, delta activity in the right hemisphere exceeded left hemisphere activity. Average delta activity was significantly more pronounced in the left hemisphere before and after training (for the pre-measurement the main effect HEMISPHERE was F(1,54) = 55.35, p < 0.0001; for the post-measurement, F(1,54) = 46.55, p <0 .0001: Figure 2).

**Figure 3:**
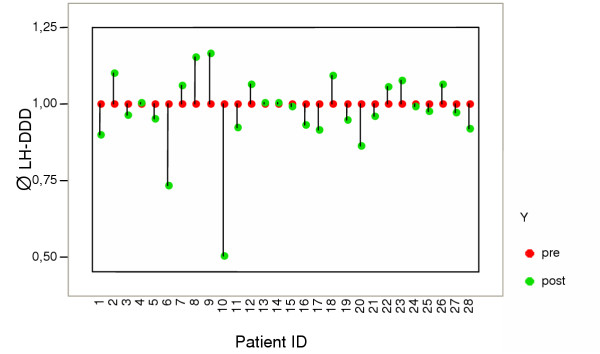
**Direction of change of DDD after therapy in the left hemisphere. **Bidirectional change in delta dipole density (DDD) after therapy. As hypothesized, we observed a decrease in DDD in 16 patients after therapy, while there was an increase in 12 patients (Note: pre-DDD scaled to 1).

Twelve patients showed an increase in left hemisphere delta activity after training, while a decrease occurred in sixteen patients (Figure 3). This diverging pattern became evident in the non-significant interaction TIME*HEMISPHERE (F(1,54) < 1). An increase in delta activity of the left hemisphere tended to covary with a longer amount of time since the lesion (F(1,26) = 3.69, p = 0.06).

### Changes in DDD relative to improvement of langauge functions

**Figure 4:**
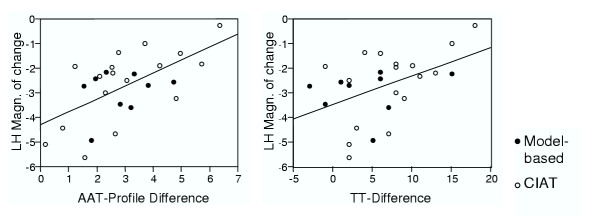
**Magnitude of change in DDD relative to changes in language measures. **Positive correlation between the "Magnitude of change" ((|T_2_-T_1_|)) in left hemispheric delta dipole density (DDD) and measures of language functions (Difference T_2_-T_1_, t-scores of AAT profile and Token Test). y-axis: data scaled by average delta activity of each patient and log-transformed. CIAT refers to patients treated according to the principles of Constrained-Induced aphasia therapy, MB refers to patients treated according to the principles of model-based aphasia therapy.

"Magnitude of change" in the left hemisphere was more pronounced in those patients who displayed significant improvement in at least one subtest of the AAT (N = 19) compared to patients with minor improvements (in at least one subscale) or no improvements (N = 9; F(1,26) = 4.95, p < 0.05). Magnitude of change of left-hemispheric delta activity varied significantly with improvements in language functions (AAT profile: r = .60, p < 0.002; Token Test: r = .46, p <0 .02, Figure 4), while there was no correlation between right hemisphere magnitude of change and language measures (AAT profile: r = .-0.07; Token Test: r = 0.01).

## Discussion

The present results provide further evidence that Abnormal Slow Wave Activity Mapping (ASWAM) discloses generators of abnormal slow waves. The mapping of abnormal slow wave activity on to brain structures allows for the identification of areas that are active, but not capable of normal function. This is, to the best of our knowledge, the first report of a re-test after controlled neuropsychological/-linguistic intervention within the same subjects. The comparison between the two measurements indicates a high reliability of peak locations in left hemispheric perilesional areas, even though successful training modified this activity in magnitude and spatial distribution. In almost all patients, the region surrounding the structural lesion continuously and reliably produced abnormal slow waves, whereas only very few patients presented slow wave activity distant from the structurally confirmed lesion. The amount of perilesional slow wave activity was markedly altered in patients who had improved after training, and the magnitude of this change was related to the changes in language functions.

Substantial functional improvements were achieved even in chronic aphasic states by shaping procedures, constraint of non-verbal communication and massed practice. This replicates and extends the findings of Pulvermüller et al. [25]. The present results further suggest that similar improvements can be achieved irrespective of the particular training procedures (a comparable improvement occurred in the model-based group). Both strategies might produce their effects – at least in part – by reorganizing brain regions next to a lesion. Following Liepert et al. [28], who demonstrated with transcranial magnetic stimulation that constraint-induced (CI) movement training of the arm expanded the area of the brain involved in generating activity in the muscles of the hand, we might assume a similar mechanism for the presently observed language improvements after intensive speech and language training in aphasics, namely an increased number of increasingly functional areas.

In contrast, some patients, though displaying language improvement after training, exhibited an increase of delta activity in the vicinity of the lesion. One explanation for this might be that the functional capabilities of the affected brain area remain disturbed, with no further potential to be restored or re-integrated in the language 'network', and this might inhibit or impair functionally intact regions. Further segregation of these continuously dysfunctional areas from the remaining network might then lead to improved language functions and consequently to increased delta activity. This hypothesis is supported by the correlation between language improvements and either decrease *or *increase of delta activity in perilesional areas. Moreover, the increase of delta activity was related to longer duration of disease. In most of the patients, re-integration of 'spared' brain areas into the language network should be completed in time (in patients exhibiting an increase of slow wave activity, the time-since-lesion averaged 55.4 ± 39.3 months, compared to 35 ± 13.9 months in patients exhibiting a decrease). Therefore, "dysfunctional" delta activity might be related, at least in a subgroup of chronic patients, to functionally more favorable outcomes. The increase of delta activity might be explained by reduced reciprocal exchange of information within functionally intact and permanently impaired network components.

## Conclusions

Compared to the more conventional procedures for aphasia treatment in the chronic stage, the present training involved an intense use of language capabilities and a restraining of alternative, non-verbal methods of communication. In our opinion, any training that encourages speech production several hours a day over several days has the potential to be efficacious. Massed practice is likely to produce activity-dependent cortical reorganization, found to result from CI-movement therapy [28-31]. It is also presumed to be the basis for a long-term increase in the amount of use of the more-affected extremity and of improved language functions following short-term intensive training.

## Methods

### Subjects

Twenty-eight patients suffering from chronic aphasia participated in the training (14 females, mean age 55 years, range 35–80 years; see Table 1 for clinical data). All patients were right-handed before brain injury, as assessed with the Edinburgh inventory [32]. In 20 patients, aphasia resulted from left-hemispheric ischemic stroke; in 8 patients it resulted from a hemorrhage affecting left-hemispheric areas. All patients were in a chronic state as defined by a time-since-lesion > 12 months. The average duration of the time-since-lesion was 43.78 months (range 12–156 months). Structural whole-head MRI was available in 26 patients and the scans were performed within the two week training period. For the other 2 patients, a left hemisphere lesion was verified by inspection of earlier MRI examination.

Prior to training, aphasia was diagnosed according to guidelines of the Aachen Aphasia Test [27], and aphasic syndromes were classified as Wernicke (N = 4), Broca (N = 13), amnesic (N = 2) and global aphasia (N = 3). Six patients could not be classified according to the 4 syndromes given on the basis of the AAT. Aphasia was evaluated as mild (N = 11), moderate (N = 16), or severe (N = 1). Patients were recruited from the local rehabilitation centre (Kliniken Schmieder Allensbach & Konstanz) or from self-help groups, or were referred by neurologists and speech therapists.

### Design and procedure

Since this report focuses on changes in slow wave activity, principles of speech and language training and results will only be summarized (a detailed description will be provided elsewhere). Training took place 3 hours/day for 10 consecutive days and included (for 18 patients) language exercises with increasing levels of difficulty [25] or (for 10 patients) model-based intervention (training based on the patients' functional deficit, with the main aim of gradually improving spoken word production).

The patients received only language therapy during the two-week training period to ensure that changes in slow wave activity were not induced by improvement of potential comorbid neurological impairment (e.g. hemiplegia). Language function was evaluated by two sensitive measures of change of aphasia severity: the profile score and the Token Test of the Aachen Aphasia Test [27]. Tests were administered by trained psychologists or speech therapists one day before the onset of training and one day after the completion of training. Language function improved significantly after training in both groups (see results) regardless of the type of training (TREATMENT*TIME interaction for AAT profile: F(1,26) = .72, p > 0.3; for Token Test: F(1,26) = 3.59, p= 0.07), therefore data from the two groups were pooled for ASWAM. Training groups did not differ significantly with respect to age or time-since-lesion.

### Data acquisition and analysis

Using a 148-channel whole-head neuromagnetometer (MAGNES™ 2500 WH, 4D Neuroimaging, San Diego, USA), MEG-measurements were collected twice: once on the day before training and once on the day after training. MEG was measured in a 5-minute resting period, during which subjects were asked to relax while staying awake, and to not engage in any specific mental activity. MEG recordings were obtained in a supine position. Subjects were asked to fixate a colored mark on the ceiling of the magnetically shielded room throughout the recording in order to avoid eye- and head-movement. A video camera installed inside the magnetically shielded room allowed for a monitoring of the subject's behavior and compliance throughout the experiment. Written informed consent was obtained from subjects prior to each MEG-session and the study was approved by the ethics committee of the University of Konstanz.

The fiducial points, coils, and head shape were digitized with a Polhemus 3Space^® ^Fasttrack prior to each measurement. The subject's head position relative to the pickup coils of the sensor was estimated before and after each measurement. MEG was recorded with a sampling rate of 678.17 Hz, using a 0.1–200 Hz band-pass filter. For artifact control, eye movements (EOG) were recorded from four electrodes attached to the left and right outer canthus and above and below the right eye. The electrocardiogram (ECG) was monitored via electrodes attached to the right collarbone and the lowest left rib using a Synamps amplifier (NEUROSCAN^®^).

### Data reduction and analysis

Data were reduced by a factor of 16 and digitally filtered for the delta (1.5–4.0 Hz) frequency band using a digital band pass filter (Butterworth filter of the order 6). Artifact-free time segments were determined by visual inspection. Single equivalent current dipoles were fitted for each time point in the selected artifact free segments (distance of time points 24 ms.). Five non-overlapping channel groups over left, right, center, anterior, posterior regions were chosen for dipole modeling. A homogeneous sphere, which gives the best least-squares fit to the digitized patient's headshape below the selected sensors, served as a model for the volume conductor.

Dipole fit solutions at time points satisfying the following requirements were accepted: (1) a dipole moment (q) of 10 nAm < q < 100 nAm; (2) a goodness of fit (GOF) greater than 0.90. These restrictions should ensure that neither artifacts nor small amplitude biological noise would affect the results, and that only dipolar fields that were generated by focal sources were analyzed. Each data-set was divided into 1000 voxels, each of 20 mm^3^, using the AFNI-to3d-software (AFNI-Analysis of functional neuroimages [33]). For each patient, the percentage of dipoles in the delta frequency band per second in each voxel was z-transformed and statistically compared to the dipole density distribution of a group of 25 healthy controls, which were considered a 'norm' group [16].

Whole brain magnetic resonance images (TR = 19, TE = 5,6, Flip angle = 30°, FOV = 256 mm, 1 mm isotropic resolution) were acquired within the 2-week training period across a 256 mm slab from each subject using a Philips Gyroscan 1.5 Tesla scanner (Philips Medical Systems, Gyroscan ACS-T). MRIs were aligned to the coordinates of the MEG according to anatomical landmarks, coil positions and head shape information using the AFNI software.

### Focal abnormal slow wave activity and its changes after training were evaluated by the following measures

#### Maximum delta activity

A spatial clustering algorithm (FWHM, Filter Width Half Maximum, 60 mm) was applied to smooth the data. Maximum activity, i.e. the voxel with the highest percentage of delta dipoles, was determined using the AFNI subroutine 3dExtrema. The localization of this maximum was determined on the x- (medial-lateral), y- (anterior-posterior) and z- (inferior-superior) plane for the two measurements (pre- & post-training).

#### Hemisphere-specific areas of high delta dipole density

The localization of areas with high dipole densities (adjacent clusters of voxels) within each hemisphere was analyzed by applying a narrow filter of FWHM 20 mm to the original data. A narrow filter was used to minimize the influence of more distant voxels with low dipole density on the areas of higher density. First, the voxel with maximum activity was determined for each hemisphere in each patient. By setting thresholds according to the following criteria, voxels with high dipole density were extracted and averaged for each hemisphere:

a. Only voxels with z-values within one standard deviation below the maximum of each patient were considered for averaging within each hemisphere.

b. If the z-value was smaller than 2 standard deviations, voxels were not considered.

c. If the peak density was below 2 SD, an iterative process was initiated. Thresholds were lowered until at least one voxel became apparent where the dipole densities were different before and after training.

#### DDD changes relative to improvement of language functions

A measure of the "Magnitude of change" was determined to evaluate changes in dipole density relative to changes in language functions pre- and post-treatment. The averaged (absolute) intensity of delta dipoles in voxels above threshold (in each hemisphere and each patient) was scaled by dividing the magnitude of change in each hemisphere (|T_2_-T_1_|) by the mean of both hemispheres before and after training ((T_1left_+T_1right_+T_2left_+T_2right_)/4). This number was then log transformed (to ensure a Gaussian distribution with respect to statistical tests) and submitted to statistical analyses. Two patients were excluded from this final analysis (12: predominantly right hemispheric delta, and 20: shift in lateralization).

#### Statistics

Changes in AAT test scores across the two assessments were verified by two-tailed t-tests. Stability of the location of the maximum delta activity was verified by correlation coefficients (Spearman's Rho) for the posterior-anterior, medial-lateral, and inferior-superior axes. Differences in thresholds and average DDD between hemispheres (for both measurements) were verified by means of analysis of variance (ANOVA), as were differences between patients that exhibited an increase or decrease of DDD concerning time-since-onset. Changes in language functions (Token Test, profile score AAT) relative to changes in DDD-magnitude were evaluated by Pearson correlation.

## Authors' contributions

MM, TE and BR participated in the design of the study. MM was responsible for conducting the study, performed data analysis and drafted the manuscript. TE and BR participated in the discussion and general conclusions. CW provided knowledge of data analysis and wrote most of the scripts used for data analysis. DD and GB conducted therapy, assisted in collecting the data, and provided experience of therapeutic issues.

## Figures and Tables

**Table 1:** Relevant clinical and demographic parameters of the training groups (Constraint-Induced aphasia therapy, CIAT; Model-based aphasia therapy, MB).

ID	Group	Sex	Age	Onset (months)^1^	Etiology	Syndrome	Severity
1	CIAT	female	35	33	hemorrhagic	Not-class.	mild
2	CIAT	female	53	32	hemorrhagic	Broca	mild
3	CIAT	male	51	13	ischemic	Wernicke	moderate
4	CIAT	male	69	33	ischemic	Wernicke	mild
5^2^	CIAT	female	70	38	hemorrhagic	Wernicke	moderate
6	CIAT	male	51	29	hemorrhagic	Broca	moderate
7	CIAT	female	47	54	hemorrhagic	Not-class.	moderate
8	CIAT	female	67	42	ischemic	Amnesic	mild
9	CIAT	male	49	92	ischemic	Broca	moderate
10	CIAT	male	41	46	hemorrhagic	Not-class.	mild
11	CIAT	male	66	26	ischemic	Not-class.	moderate
12	CIAT	male	39	56	ischemic	Amnesic	mild
13	CIAT	male	36	12	ischemic	Broca	mild
14	CIAT	female	47	87	ischemic	Broca	moderate
15	CIAT	female	53	50	ischemic	Broca	moderate
16	CIAT	female	80	23	ischemic	Wernicke	mild
17	CIAT	male	47	29	ischemic	Broca	moderate
18	CIAT	female	36	32	ischemic	Global	moderate
19	MB	male	59	39	hemorrhagic	Not-class.	mild
20	MB	male	37	36	ischemic	Broca	moderate
21	MB	female	49	28	ischemic	Global	severe
22	MB	male	57	40	ischemic	Broca	mild
23	MB	male	62	156	ischemic	Broca	moderate
24^2^	MB	female	76	13	ischemic	Global	moderate
25	MB	male	66	64	ischemic	Broca	moderate
26	MB	female	65	29	hemorrhagic	Broca	moderate
27	MB	female	47	53	ischemic	Broca	mild
28	MB	female	75	40	ischemic	Not-class.	moderate
N = 28	18 CIAT 10 MB	14 ♀/14 ♂	54,6	43,8	20 I, 8 H		N = 26

^1 ^MRI was acquired within the two-week training period. ^2 ^For patients 5 and 24, MRI could not be acquired within the training period. Lesions of the left hemisphere were verified by clinical case report forms obtained from earlier clinical presentation (for both patients, MRI was performed immediately after the insult).
